# INTERVENTIONS TO PREVENT ELDER MISTREATMENT WITH FAMILY CAREGIVERS OF PERSONS LIVING WITH DEMENTIA

**DOI:** 10.1093/geroni/igae098.0932

**Published:** 2024-12-31

**Authors:** Kylie Meyer, Jeanine Yonashiro-Cho, Frank Puga

**Affiliations:** Chair; Co-Chair; Discussant

## Abstract

Persons living with Alzheimer’s disease and related dementias are among the most vulnerable to elder mistreatment and neglect (EM). Family caregivers, who often enter this role with minimal preparation or support, are among the most likely to engage in EM. In this symposium, we describe novel interventions to prevent EM by family caregivers of persons living with dementia. In the first talk, Dr. Jablonski will describe the Coaching Dementia Caregivers to Master Care-Resistant Behavior (CuRB-IT) intervention, which uses 1:1 Zoom coaching to help caregivers respond to care-resistant behaviors. This includes a discussion of preliminary findings on intervention acceptability. Next, Dr. Meyer will provide results from a pilot study of the Knowledge and Interpersonal Skills to Develop Enhanced Relationships (KINDER) intervention, a group-based psychoeducation intervention to prevent EM by focusing on the care relationship. Dr. Wilber will then discuss secondary results from the double-blinded trial of the Comprehensive Older Adult and Caregiver Help (COACH), which was informed by family violence interventions. She will discuss the perplexing finding wherein risk factors for abuse were largely unaffected by the COACH intervention despite the elimination of mistreatment in this arm. Lastly, Dr. Yonashiro-Cho will describe basic behavioral research drawn from the Better Together study to inform a culturally tailored version of the EM prevention program that addresses the needs of Asian American and/or Pacific Islander family caregivers. These studies represent multiple stages of intervention development, and each addresses an emerging area of research to prevent elder mistreatment within a dementia care context.

